# Primary Tumor-Secreted Lymphangiogenic Factors Induce Pre-Metastatic Lymphvascular Niche Formation at Sentinel Lymph Nodes in Oral Squamous Cell Carcinoma

**DOI:** 10.1371/journal.pone.0144056

**Published:** 2015-12-02

**Authors:** Naohiro Wakisaka, Yasuhisa Hasegawa, Seiichi Yoshimoto, Kouki Miura, Akihiro Shiotani, Junkichi Yokoyama, Masashi Sugasawa, Makiko Moriyama-Kita, Kazuhira Endo, Tomokazu Yoshizaki

**Affiliations:** 1 Division of Otolaryngology, and Head & Neck Surgery, Kanazawa University, Kanazawa, Ishikawa, Japan; 2 Department of Head and Neck Surgery, Aichi Cancer Center, Nagoya, Aichi, Japan; 3 Department of Head and Neck Oncology, National Cancer Center Hospital, Chuo-ku, Tokyo, Japan; 4 Department of Head and Neck Oncology and Surgery, International University of Health and Welfare Mita Hospital, Minato-ku, Tokyo, Japan; 5 Department of Otolaryngology-Head & Neck Surgery, National Defense Medical College, Tokorozawa, Saitama, Japan; 6 Department of Otorhinolaryngology, Juntendo University Faculty of Medicine, Bunkyo-ku, Tokyo, Japan; 7 Department of Head and Neck Surgery and Otolaryngology, Saitama Medical University International Medical Center, Hidaka, Saitama, Japan; China Medical University, TAIWAN

## Abstract

**Objectives:**

The objectives of this study were to evaluate the formation of lymphvascular niches in lymph nodes of patients with oral squamous cell carcinoma (OSCC), and investigate the roles of lymphangiogenic and angiogenic factors, such as vascular endothelial growth factor (VEGF)-A, VEGF-C, and VEGF-D, expressed in the primary tumors.

**Materials and Methods:**

Forty-four patients with previously untreated clinically late T2 or T3 OSCC of cN0 were evaluated for primary tumors and 166 sentinel lymph nodes (SLNs). Primary tumors were immunohistochemically analyzed for expressions of VEGFs. Densities of lymphatic vessels (LVD_podoplanin_) and high endothelial venules (HEVD) in the SLNs were also calculated using antibodies for each marker, podoplanin and MECA-79, respectively.

**Results:**

In 25 patients, all lymph nodes were metastasis-negative, whereas, in 19 patients, metastasis was positive for at least one lymph node (either at SLN, non-SLN, or nodal recurrence). From the analyses of 140 SLNs without metastasis, LVD_podoplanin_ in 50 SLNs of metastasis-positive cases was significantly higher than that in 90 SLNs of metastasis-negative cases (*p* = 0.0025). HEVD was not associated with lymph node metastasis. The patients with VEGF-A-High or VEGF-D-High tumors had significantly higher LVD_podoplanin_ than patients with their Low counterparts (*p* = 0.0233 and *p* = 0.0209, respectively). In cases with lymph node metastasis, the VEGF-D-expression score was significantly higher than in those without lymph node metastasis (*p* = 0.0006).

**Conclusions:**

These results suggest that lymph node lymphangiogenesis occurs before metastasis in OSCC. VEGF-A and VEGF-D play critical roles in this process. VEGF-D is a potential predictive marker of positive lymph node metastasis in cN0 patients.

## Introduction

Experiments focused on the biology of lymphatics were triggered by the discovery of specific lymphatic endothelium markers, such as podoplanin, lymphatic vessel endothelial hyaluronan receptor-1 (LYVE-1), and prox-1, differentiating lymphatics from blood vascular endothelium [1]. The contribution of the lymphatic system to tumor lymph node metastasis is being increasingly appreciated through studies of human cancer tissues, such as carcinoma of the breast, oral cavity, colon, and prostate as well as melanoma [2,3,4]. Vascular endothelial growth factor (VEGF)-C and VEGF-D were identified as tumor-derived secretory factors (TDSFs), being predominantly lymphangiogenic, *via* the VEGF receptor 3 (VEGFR3), which is expressed in lymphatic endothelial cells [5]. In addition to VEGF-C and VEGF-D, overexpression of VEGF-A also leads to the activation of lymphangiogenesis [6]. The functions and roles of these lymphangiogenic factors have been investigated with regard to peritumoral and intratumoral tumor lymphangiogenesis. However, the experimental reports are limited on the molecular determinant of lymph node lymphangiogenesis in human cancer.

High endothelial venules (HEVs) are specialized venules that are lined by plump endothelial cells. HEVs occur in secondary lymphoid organs, except the spleen, and are the main sites of lymphoid entry from the blood. The antibody MECA-79, which has been widely used to characterize HEVs, binds to 6-sulpho sialyl Lewis X on core 1 *O*-glycans, a unique feature of HEV sialomucins [7,8]. Recently, it was shown that HEVs are frequently found in the stromas of solid tumors (such as melanomas and breast, colon, lung, and ovarian carcinomas) [7]. A high density of these tumor HEVs is associated with high levels of infiltration by B and T cells (including CD8^+^ cytotoxic T cells), as well as with a favorable clinical outcome in breast cancer patients [9].

Neck lymph node involvement is considered the most important adverse prognostic factor in head and neck cancers, including oral squamous cell carcinoma (OSCC) [10]. Metastasis to cervical lymph nodes occurs in approximately 30% of patients with early OSCC and is associated with regional recurrence and a poor outcome [11,12]. Although close observation (i.e., watchful waiting) remains an option, most clinicians favor excision of the regional lymphatics at the time of resection of the primary cancer for accurate staging. To date, the sentinel lymph node (SLN) concept has been extensively validated in OSCC as well as melanoma and breast cancer [3,4,13]. SLN biopsy allows the surgeon to identify and excise targeted lymph nodes that drain the site of a primary malignancy [14,15]. In practice, if the SLNs are negative, a morbid regional lymph node dissection can be avoided. Although some reported the overall sensitivity of SLN biopsy > 90% in OSCC, it is not yet possible to say whether the results of SLN identification are consistent and reliable [10,16,17].

Paget proposed the “seed and soil” hypothesis, over a century ago, wherby the “seed” (tumor cells) selectively colonizes the “soil” of distant organs with an environment favorable for survival and proliferation [18]. TDSFs from the primary tumor promote the mobilization and recruitment of bone marrow-derived cells that interact with the local stroma and extracellular matrix at secondary organs, to help create a microenvironment, termed a pre-metastatic niche, suitable for colonization prior to tumor cell dissemination [19]. In some models, tumor-secreted lymphangiogenic factors promote the enlargement of lymphatic networks inside the SLN, known as sinusoidal hyperplasia, and may also affect the lymphatic vasculature [2]. The remarkable enlargement of sinusoidal lymphatic endothelium might facilitate tumor cell transport to the lymph nodes, and potentially contribute to the migration, residence, and/or survival of metastatic tumor cancer stem cells by inducing a specific tumor microenvironment, lymphvascular niche [6,20]. On analyzing regional lymph nodes of tongue cancer, Lee et al. reported that the density of dilated HEVs was significantly higher in patients with established metastasis in their lymph nodes [21]. Therefore, from the perspective of pre-metastatic niche formation, it is crucial to understand the changes of the lymphvascular system, such as lymphatic vessels and HEVs, in the receiving SLN.

The objectives of this study were to evaluate the SLN lymphvascular system in OSCC patients prior to metastasis, and to investigate the roles of tumor-derived lymphangiogenic and angiogenic factors, such as VEGF-A, VEGF-C, and VEGF-D, in SLN lymphvasculogenesis. The study demonstrates that tumor-induced SLN lymphangiogenesis occurs before metastasis in OSCC, and that tumor-derived VEGF-A and VEGF-D play significant roles in that process.

## Materials and Methods

### Patients and tissues

Fifty-seven patients from seven institutions, previously untreated clinically late T2 or T3 OSCC with negative necks diagnosed by physical examination and imaging evaluation with computed tomography (CT) or magnetic resonance imaging (MRI), who had undergone surgical treatment, including SLN biopsy, between 2009 and 2011, were enrolled onto the present retrospective study. Among them, written informed consent, according to the approval of ethics committee of Kanazawa University (2012–004), was obtained from forty-six patients. Patient records/information was anonymized and de-identified prior to analysis. Written informed consent was given by all of the participants for their clinical records to be used in this study. Two patients with neck recurrence accompanied by primary recurrence were excluded from this retrospective analysis, because of the possibility of *de novo* lymph node metastasis from the residual primary tumors. Eventually, we evaluated 44 primary tumor and 166 SLN tissues from 44 patients.

### Intraoperative SLN biopsy, and neck dissection

The radioactive tracer used was 74 MBq of technecium 99m (99m-Tc) phytate, which was injected submucosally around the primary tumor at four points the day before surgery [17]. Based on fusion images of single photon emission computed tomography and CT, SLNs were extracted intraoperatively using a handheld gamma probe and sent for pathologic analysis. When a metastasis-positive SLN was found, a unilateral supraomohyoid neck dissection (level I, II, and III) on the affected side with addition of corresponding level, if necessary, was performed. The SLNs and all other dissected lymph nodes were examined for disease. Frozen sectioning was used intraoperatively as rapid analysis in all cases. The attending pathologist examined SLN sections cut from approximately 2-mm thickness blocks with hematoxylin-eosin stain. For postoperative pathological diagnosis, 4-μm sections from each 2-mm thickness block were examined with hematoxylin-eosin stain and immunohistochemical stain for pan-cytokeratin. The same pathologist examined the remaining neck lymph nodes in a single representative cross-section.

### Immunohistochemical analysis

The surgical specimens including primary tumors and SLNs were fixed in a 10% formalin solution and embedded in paraffin. Consecutive 3-μm sections were cut from each block. Immunohistochemical staining was performed as described previously [22]. The following primary antibodies were used: mouse-derived monoclonal antibody for podoplanin (dilution 1:100; Dako, Carpinteria, CA, USA), rabbit-derived polyclonal antibody for VEGF-A (dilution 1:200; Santa Cruz Biotechnology, Dallas, TX, USA), rabbit-derived polyclonal antibody for VEGF-C (dilution 1:100; Invitrogen, Carlsbad, CA, USA), mouse-derived monoclonal antibody for VEGF-D (dilution 1:100; R&D Systems, Minneapolis, MN, USA), mouse-derived monoclonal antibody for pan-cytokeratin (dilution 1:100; Dako, Carpinteria, CA, USA), goat-derived polyclonal antibody for VEGFR3 (dilution 1:50; R&D Systems, Minneapolis, MN, USA), and rat-derived monoclonal antibody for MECA-79 (dilution 1:100; Santa Cruz Biotechnology, Dallas, TX, USA). Diaminobenzidine tetrahydrochloride was used as a chromogen, and the sections were counterstained with hematoxylin. The specificities of the staining were confirmed using non-immune serum instead of the primary antibody as a negative control. Two investigators (N.W. and M.M-K.) who had no prior knowledge of the clinicopathological findings assessed the lymph nodes for lymphatic vessels highlighted by lymphatic markers, podoplanin and VEGFR3 stainings, and HEVs highlighted by MECA-79 staining, and primary sites for expressions of VEGF-A, VEGF-C, VEGF-D, and VEGFR3. Each lymph node was also analyzed for pan-cytokeratin expression to detect metastatic tumor cells.

To evaluate the densities of lymphatic vessels and sinuses, and HEVs in SLNs, imaging analysis was performed using Adobe Photoshop CS3 Extended^®^ (Adobe Systems, San Jose, CA, USA). After identifying the site of the most aggressive focus of lymphvasculogenesis detected by each antibody for lymphatic vessels and HEVs, an image of the region was obtained as a 200x magnification field (1.1 mm^2^). The lymphatic vessel density (LVD) and HEHEV density (HEVD) were obtained by dividing the measured area of immunostained vessels by the input region area (i.e., a size equivalent to the 200x magnification high-power field) for each SLN. LVD evaluated by podoplanin or VEGFR3 immunostaining was termed LVD_podopalnin_ or LVD_VEGFR3_, respectively.

Positive VEGF-A, VEGF-C, VEGF-D, and VEGFR3 stainings at the primary sites were semi-quantitatively assessed by multiplying the staining intensity [none (0), weak (1), moderate (2), or strong (3)] by the rate of tumor cells stained [0 (0%), 1 (1–10%), 2 (11–20%), 3 (21–30%), 4 (31–40%), 5 (41–50%), 6 (51–60%), 7 (61–70%), 8 (71–80%), 9 (81–90%), or 10 (91–100%)]. The median staining scores were selected as cut-off values to categorize the tumor into High and Low-expressing primary tumors for VEGFs and VEGFR3 stainings.

### Staistical analysis

IBM SPSS Statistics, version 19 (IBM, Armonk, New York, USA), was used for data analysis. The clinicopathological parameters in relation to LVDs and HEVD were analyzed using the Mann-Whitney *U*-test. The development of nodal metastasis and its correlations with clinicopathological parameters were analyzed with Fisher’s exact test. For statistical analysis, patients with positive SLN metastasis, non-SLN metastasis, or nodal recurrence were defined as lymph node metastasis-positive cases. A *p*-value of 0.05 or less was considered significant.

## Results

### Patient Characteristics

**Fig 1:**
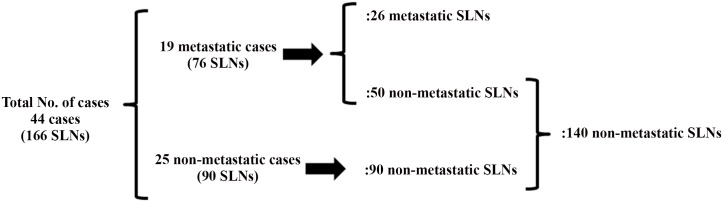
The detailed distribution of the 166 sentinel lymph nodes (SLNs) examined in this study. The typical patient had 3–4 SLNs in this study. 26 metastatic and 50 non-metastatic SLNs are from 19 metastatis-positive cases. 90 non-metastatic SLNs are from 25 metastasis-negative cases. SLNs, sentinel lymph nodes.

The detailed characteristics of the forty-four patients with OSCC studied are described in Table 1. Thirty-eight cases were late T2 (tumor more than 3 cm but not more than 4 cm in its greatest dimension) (c late T2N0M0) and six cases were T3 (cT3N0M0). The patients comprised 33 males and 11 females, with ages ranging from 30 to 85 years (median, 59 years). The median follow-up among surviving patients was 37.5 months (from 30 to 49 months). We analyzed all surgically extracted lymph nodes including 166 SLNs from the forty-four patients. The typical patient had 3–4 SLNs in this study, whereas it might be less common to have this many SLNs in other tumor types [3,4,16,17]. The detailed distribution of the 166 SLNs is shown in Fig 1. In 25 patients, all lymph nodes including the 90 SLNs were metastasis-negative. In the 19 patients with metastasis, 26 SLNs were metastasis-positive and 50 SLNs were metastasis-negative. Thus, among the 166 SLNs, 140 SLNs were metastasis-negative and 26 were metastasis-positive. In the 19 patients, metastasis was positive for at least one lymph node (either at SLN, non-SLN, or nodal recurrence), among which metastasis was detected only in SLNs in 13 cases. In 4 cases, SLN metastasis was accompanied by non-SLN metastasis (2 cases) or nodal recurrence (2 cases), respectively. In 2 cases without SLN metastasis, non-SLN metastasis was found with or without nodal recurrence.

**Table 1:** Characteristics of patients.

Characteristics	Value
**Total No. of Cases**	44
**Age, y**	
Mean ± SD	57.455 ± 15.205
Range	30–85
**Sex, n (%)**	
Male	33 (75.0)
Female	11 (25.0)
**Primary tumor site, n (%)**	
Oral tongue	37 (84.1)
Mouth floor	3 (6.8)
Gingiva	3 (6.8)
Buccal mucosa	1 (2.3)
**T-status, n (%)**	
Late T2	38 (86.4)
T3	6 (13.6)
**Depth of invasion, mm**	
Mean ± SD	10.341 ± 8.029
Range	1.0–41.0
**Lymph node metastasis (SLN, non-SLN, or nodal recurrence), n (%)**	
Non-metastatic cases	25 (56.8)
Metastatic cases	19 (43.2)
**Treatment outcome**	
Recurrence, n (%)	
Primary	3 (6.8)
Regional	3 (6.8)
Status, n (%)	
No evidence of disease	40 (90.9)
Died of disease	4 (9.1)
Died from other cause	0 (0.0)

SLN, sentinel lymph node; SD, standard deviation.

### Detection of lymphatic vessels and sinuses, and HEVs in SLNs (Fig 2)

**Fig 2:**
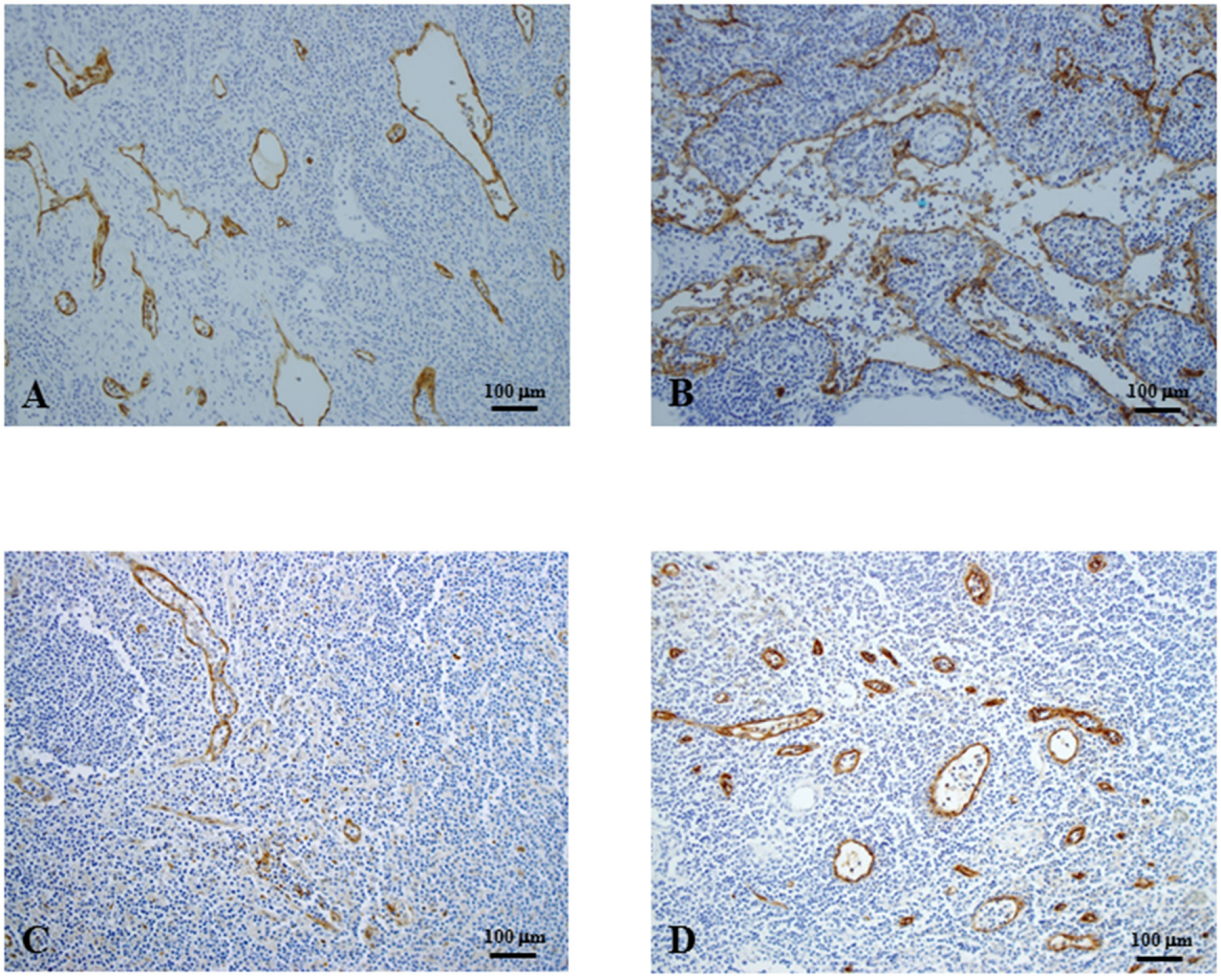
Immunohistochemistry of sentinel lymph nodes (original magnification, x100). A, B, Podoplanin antibody for lymphatic endothelial cells. Lymphatic vessels and sinuses highlighted by podoplanin staining were scattered (A) or accumulated (B) in the stroma of lymphatic tissues. C, VEGFR3 antibody for lymphatic endothelial cells. Lymphatic vessels and sinuses were detected as scattered lumens in a similar staining pattern to podoplanin staining. D, High endothelial venules detected by MECA-79 antibody were round, and some endothelial cells were effaced and lumens were wide and fusiform. VEGFR3, vascular endothelial growth factor 3.

The antibody to podoplanin specific for lymphatic endothelium detected lymphatic vessels and also recognized expanded lymphatic sinuses (Fig 2A and 2B). The markedly increased and enlarged lymphatic vessels and sinuses were distributed throughout the cortex and medulla of the lymph nodes. The antibody to VEGFR3 also showed dilated lymphatic vessels (Fig 2C). The morphological features of MECA-79-positive HEVs were similar to the lymphatic vessels. Lumens of HEVs were found to be round, but some endothelial cells were effaced and lumens were wide and fusiform (Fig 2D).

### VEGF-A, VEGF-C, VEGF-D, and VEGFR3 expressions at the primary tumors (Fig 3)

**Fig 3:**
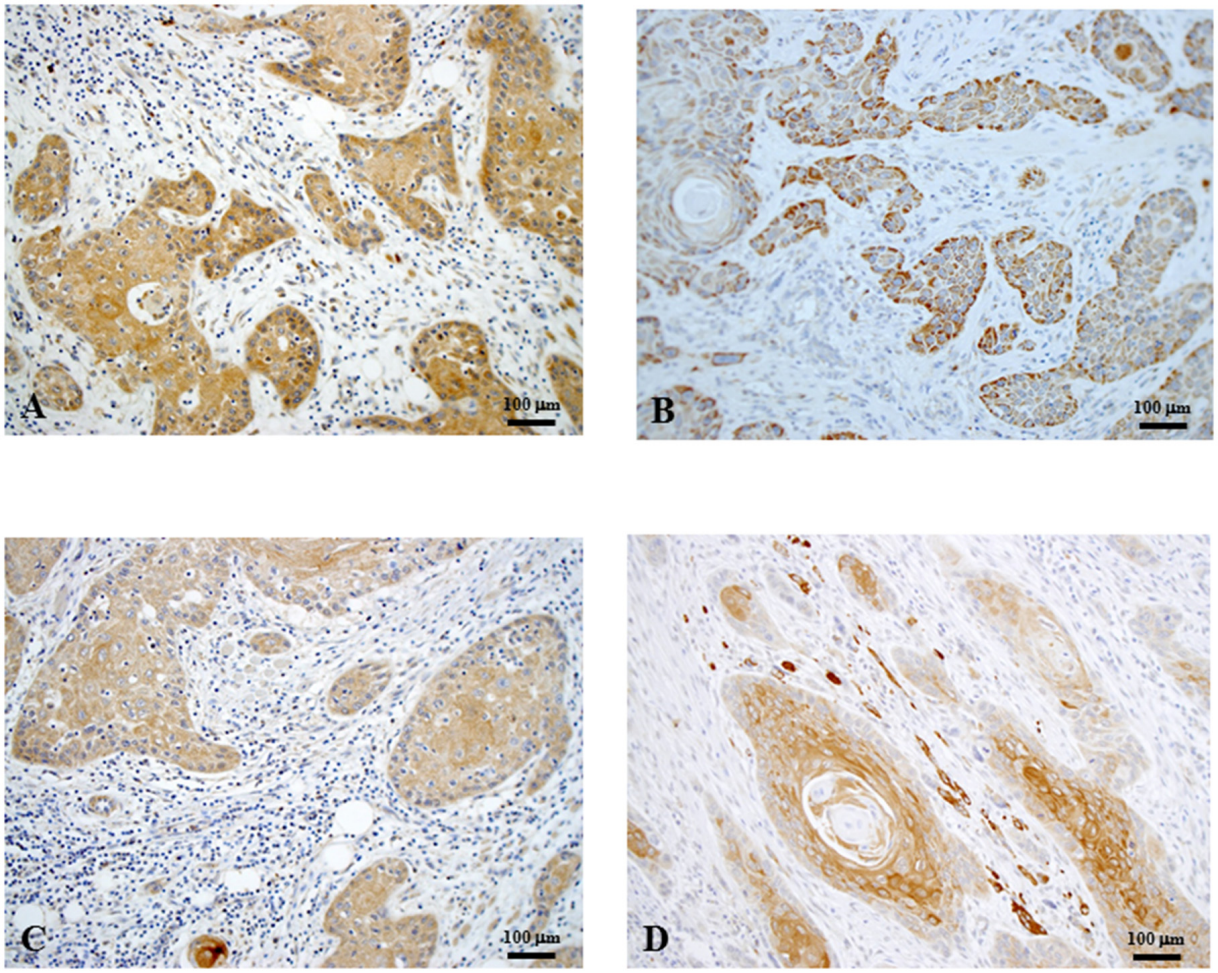
Representative images of VEGF-A (A), VEGF-C (B), VEGF-D (C), and VEGFR3 (D) immunohistochemical staining in primary oral squamous cell carcinoma (original magnification, x100). VEGF-A, VEGF-C, and VEGF-D were mainly immunolocalized in the cytoplasm of tumor cells. VEGFR3 was localized in the cytoplasm and/or at the membrane of tumor cells. VEGF, vascular endothelial growth factr; VEGFR3, VEGF receptor 3.

VEGF-A, VEGF-C, and VEGF-D were localized in the cytoplasm and occasionally on the membrane of OSCC tumor cells (Fig 3A, 3B and 3C). VEGF-A was detected in some inflammatory cells around carcinoma nests. Occasionally, VEGF-D was detected in vascular endothelial cells near the carcinoma nests. According to the criteria used for the immunohistochemical staining of these proteins, expressions of VEGF-A, VEGF-C, and VEGF-D were categorized as High in 23, 22, and 23 of the 44 cases, respectively.

VEGFR3 was immunolocalized in the cytoplasm and/or at the cell membrane of tumor cells (Fig 3D). VEGFR3 expression was categorized as High in 22 cases.

### Association of LVD_podoplanin_, LVD_VEGFR3_, and HEVD with lymph node metastasis

Association of LVD_podoplanin_, LVD_VEGFR3_, and HEVD with lymph node metastasis is shown in Table 2. Again, the detailed distribution of the 166 SLNs is shown in Fig 1. The LVD_podoplanin_ in the 26 metastatic lymph nodes was significantly higher than that in 140 non-metastatic lymph nodes (0.293±0.237 vs. 0.189±0.152, *p* = 0.0459). The LVD_podoplanin_ in 76 SLNs of metastasis-positive cases was significantly higher than that in 90 SLNs of metastasis-negative cases (0.258±0.203 vs. 0.159±0.123, *p* = 0.0006). In addition, from the analyses of 140 SLNs without metastasis, we found that the LVD_podoplanin_ in 50 SLNs of metastasis-positive cases had significantly higher LVD_podoplanin_ than that in 90 SLNs of metastasis-negative cases (0.242±0.181 vs. 0.159±0.123, *p* = 0.0025).

**Table 2:** Association of LVDpodoplanin, LVDVEGFR3 and HEVD with lymph node metastasis.

SLNs	No. of SLNs	LVD_podoplanin_		LVD_VEGFR3_		HEVD	
		Mean ± SD	p	Mean ± SD	p	Mean ± SD	p
Total SLNs	166	0.203 ± 0.166	N.A.	0.050 ± 0.049	N.A.	0.119 ± 0.087	N.A.
Non-metastatic SLNs	140	0.189 ± 0.152	0.0459	0.047 ± 0.047	0.0476	0.120 ± 0.094	0.3367
Metastatic SLNs	26	0.293 ± 0.237		0.070 ± 0.056		0.112 ± 0.043	
SLNs of non-metastatic cases	90	0.159 ± 0.123	0.0006	0.045 ± 0.049	0.6565	0.114 ± 0.083	0.3042
SLNs of metastatic cases	76	0.258 ± 0.203		0.058 ± 0.050		0.126 ± 0.095	
Non-metastatic SLNs	140						
SLNs of non-metastatic cases	90	0.159 ± 0.123	0.0025	0.045 ± 0.049	0.3661	0.114 ± 0.083	0.5651
SLNs of metastatic cases	50	0.242 ± 0.181		0.051 ± 0.045		0.138 ± 0.122	

N.A., not applicable; SLN, sentinel lymph node; LVD, lumphatic vessel density; HEVD, high endothelial venule density; SD, standard deviation. The subsets of SLN in relation to LVDs and HEVDwere analyzed using Mann-Whitney U-test. Patients with positive sentinel lymph node metastasis, non-sentinel lymph node metastasis, or nodal recurrence were defiened as lymph node metastasis-positive cases.

The LVD_VEGFR3_ in the metastatic SLNs was significantly higher than in non-metastatic SLNs (0.070±0.056 vs. 0.047±0.047, *p* = 0.0476). However, LVD_VEGFR3_ was similar between metastatis-positive and metastasis-negative cases with or without the inclusion of metastatic SLNs in the analyses.

The HEVD in the metastatic SLNs was similar to that in the non-metastatic SLNs. The HEVD was also similar between metastasis-positive and metastasis-negative cases with or without the inclusion of metastatic nodes in the analysis.

### The relationships between clinicopathological factors and expressions of VEGFs, and LVD_podoplanin_, LVD_VEGFR3_, and HEVD

The relationship between the expression of VEGFs in the primary tumors and LVD_podoplanin_ was examined (Table 3). The patients with VEGF-A-High tumors had significantly higher LVD_podoplanin_ than those with VEGF-A-Low tumors (0.232±0.169 vs. 0.181±0.159, *p* = 0.0233). VEGF-C expression was not associated with LVD_podoplanin_. The LVD_podoplanin_ was significantly higher in VEGF-D-High cases than in its Low counterpart (0.235±0.185 vs. 0.176±0.141, *p* = 0.0477).

**Table 3:** The relationship between clinicopathological factors and expression of VEGFs, and LVDpodoplanin, LVDVEGFR3, and HEVD.

Characteristics	No. of Cases	LVD_podoplanin_		LVD_VEGFR3_		HEVD	
		Mean ± SD	p	Mean ± SD	p	Mean ± SD	p
Total cases	44	0.203 ± 0.166	N.A.	0.050 ± 0.049	N.A.	0.119 ± 0.087	N.A.
Sex							
Male	33	0.202 ± 0.171	0.4746	0.051 ± 0.051	0.7875	0.121 ± 0.090	0.5164
Female	11	0.215 ± 0.137		0.048 ± 0.038		0.113 ± 0.078	
Age, y							
≧60	21	0.186 ± 0.147	0.1964	0.049 ± 0.043	0.9500	0.111 ± 0.077	0.8247
<60	23	0.220 ± 0.180		0.051 ± 0.054		0.125 ± 0.096	
T-status							
late T2	38	0.207 ± 0.170	0.2467	0.049 ± 0.049	0.1262	0.115 ± 0.087	0.0971
T3	6	0.177 ± 0.132		0.072 ± 0.050		0.132 ± 0.091	
Depth of invasion (mm)							
≧11	20	0.204 ± 0.153	0.6765	0.050 ± 0.042	0.4585	0.125 ± 0.084	0.1242
<11	24	0.203 ± 0.174		0.050 ± 0.054		0.114 ± 0.090	
VEGF-A expression							
High	21	0.232 ± 0.169	0.0233	0.059 ± 0.058	0.0904	0.135 ± 0.094	0.0048
Low	23	0.181 ± 0.159		0.043 ± 0.039		0.104 ± 0.077	
VEGF-C expression							
High	22	0.217 ± 0.117	0.2600	0.058 ± 0.055	0.0209	0.125 ± 0.083	0.1102
Low	22	0.184 ± 0.145		0.039 ± 0.035		0.111 ± 0.093	
VEGF-D expression							
High	21	0.235 ± 0.185	0.0477	0.055 ± 0.049	0.2316	0.116 ± 0.082	0.9539
Low	23	0.176 ± 0.141		0.046 ± 0.049		0.121 ± 0.091	

N.A., not applicable; VEGF, vascular endothelial growth factor; LVD, lymphatic vessel density; HEVD, high endothelial venule density; SD, standard deviation. The clinicopathological parameters in relation to LVDs and HEVD were analyzed using Mann-Whitney U-test.

Next, we examined the association between LVD_VEGFR3_ and expression of VEGFs in the primary tumor (Table 3). VEGF-C-High tumors had significantly higher LVD_VEGFR3_ than VEGF-C-Low tumors (0.058±0.055 vs. 0.039±0.035, *p* = 0.0209). The LVD_VEGFR3_ was similar in relation to the expression level of VEGF-A or VEGF-D in primary tumors, respectively.

HEVD was significantly higher in VEGF-A-High tumors than VEGF-A-Low ones (0.135±0.094 vs. 0.104±0.077, *p* = 0.0048). The HEVD was not associated with the expression of VEGF-C or VEGF-D in the primary tumors, respectively.

Other clinicopathological factors, such as the sex, age, T-status, and depth of invasion, were not significantly correlated with these lymphvascular densities.

### Associations of lymph node metastasis with expression of VEGFs

Associations of lymph node metastasis with expression of VEGFs are shown in Table 4. No clinical factor, such as the sex, age, T-status, or depth of invasion, was associated with the nodal status. In cases with lymph node metastasis, the VEGF-D-expression score was significantly higher than in those without lymph node metastasis (*p* = 0.0006), while the expressions of VEGF-A and VEGF-C were not related to lymph node involvement, respectively.

**Table 4:** Associations of lymph node metastasis with expression of VEGFs.

Characteristics	No. of Cases	Lymph Node Metastases		p
		Positive	Negative	
No. of Cases	44	19	25	
Sex				
Male	33	15	18	0.7315
Female	11	4	7	
Age, y				
≧60	21	9	12	>0.9999
<60	23	10	13	
T-status				
late T2	38	17	21	0.6843
T3	6	2	4	
Depth of invasion (mm)				
≧11	20	11	9	0.2227
<11	24	8	16	
VEGF-A expression				
High	21	12	9	0.1271
Low	23	7	16	
VEGF-C expression				
High	22	10	12	>0.9999
Low	22	9	13	
VEGF-D expression				
High	21	15	6	0.0006
Low	23	4	19	
VEGFR3 expression				
High	22	11	11	0.5434
Low	22	8	14	
VEGF-C & VEGFR3 expressions				
High	12	6	6	0.7350
Low	32	13	19	
VEGF-D & VEGFR3 expressions				
High	14	11	3	0.0025
Low	30	8	22	

VEGF, vascular endothelial growth factor; VEGFR3, vascular endothelial growth factor receptor 3. The development of nodal metastasis and its correlation with clinicopathological parameters were analyzed with Fisher's exact test. Patients with positive sentinel lymph node metastasis, non-sentinel lymph node metastasis, or nodal recurrence were defined as lymph node metastasis-positive cases.

VEGFR3 expression in the primary tumor cells at the primary site was also examined to clarify the role of the VEGFR3-associated signaling pathway with its ligands, VEGF-C and VEGF-D. Although VEGFR3-expression in the primary tumor cells was not associated with lymph node metastasis (*p* = 0.5434), lymph node metastasis was significantly progressed when both VEGF-D and VEGFR3-expressions were High (*p* = 0.0025).

## Discussion

Pre-metastatic niches are now widely accepted as a true biological process promoting metastatic growth [23,24]. Recent data suggest that tumor cell migration is facilitated by lymphangiogenesis, the generation of new lymphatic vessels from pre-existing lymphatics or lymphatic endothelial progenitors [25,26]. Therefore, we evaluated lymphangiogenesis in SLNs to clarify that tumor-draining SLNs show enhanced lymphangiogenesis even before cancer metastasis and they may function as a permissive “lymphatic niche” for the survival of metastatic cells [27]. Here, we demonstrated that LVD_podoplanin_ was markedly increased even in the metastasis-negative SLNs of patients with metastasis compared to those without metastasis. This suggests that lymphangiogenesis occurs even before the arrival of tumor cells in the SLNs of OSCC. Therefore, in cases of tumor-free lymph nodes, the increased lymphatic network of SLNs is a very early pre-metastatic sign and may provide a new prognostic indicator of the diseases [22]. Tumor-derived signals are transported via the lymphatics to the draining LN, where they induce localized lymphatic vessel growth [20]. Here, we investigated the expressions of lymphangiogenic factors, such as VEGF-A, VEGF-C, and VEGF-D, at the primary tumors in relation to SLN lymphangiogenesis. When the expression of VEGF-A or VEGF-D was High, LVD_podoplanin_ in SLNs was markedly increased. These results suggest that the expression of VEGF-A or VEGF-D in the primary tumors induces lymphangiogenesis in the SLNs of OSCC patients.

VEGF-C and VEGF-D specifically activate VEGFR3 on lymphatic endothelium to induce lymphatic capillary proliferation and growth [28,29]. As expected, the LVD_VEGFR3_ was significantly higher in VEGF-C-High tumors than in Low tumors. However, VEGF-D expression was not associated with LVD_VEGFR3_. These results suggest that tumor-cell-derived VEGF-C, but not VEGF-D, induces the proliferation of VEGFR3-expressing lymphatic vessels in SLNs of OSCC patients. From the analysis of 23 metastasis-negative SLNs from 10 patients, Ishii et al. showed that SLNs from patients with VEGF-C-positive tumors showed a significantly higher amount of VEGFR3 mRNA, reflecting the level of VEGFR3-positive lymphatic vessels, than those from patients with VEGF-C-negative tumors [4]. However, VEGF-D expression in the primary tumor was not correlated with the amount of VEGFR3 mRNA in the SLNs. These results are in agreement with our findings. VEGF-D may stimulate the proliferation of lymphatic endothelial cells through its receptor distinct from VEGFR3, such as VEGFR2 [30]. In the current study, although LVD_VEGFR3_ in the metastatic SLNs was significantly higher than that in the non-metastatic SLNs, LVD_VEGFR3_ was similar between metastatic and non-metastatic cases in the analysis of non-metastatic SLNs. Therefore, in the SLNs of OSCC patients, lymphangiogenesis through the VEGF-C-VEGFR3 pathway may be stimulated in the late phase of the metastatic process or after the arrival of tumor cells at SLNs, but not involved in the formation of the pre-metastatic lymphatic niche.

Little is known about the morphological changes and clinical implications of HEVs, which play an important role in recruiting lymphocytes for the generation of immune responses inside lymph nodes [31,32]. Shrestha et al. showed that B-cell-derived VEGF-A promoted lymphangiogenesis and the expansion of HEVs in lymph nodes, and then suppressed certain aspects of immune responses [33]. In the current study, VEGF-A-High tumors had significantly higher HEVD than VEGF-A-Low tumors. Thus, VEGF-A plays an important role in the increasing density of HEVs both in inflamed and cancer-associated lymph nodes. However, HEVD was not associated with lymph node metastasis, which was inconsistent with the previous report by Lee et al. [21]. They found abnormally dilated HEVs containing red blood cells in lymph nodes of metastasis-positive tongue cancer [21]. The inconsistency may be, at least in part, because of the difference in the study population. They analyzed regional lymph nodes, not SLNs, of surgically treated patients who underwent neck dissection. Nearly half of their patients were with pathologically proven lymph node metastasis, including patients with clinically evident lymph node metastasis before treatment. In contrast, the current study exclusively analyzed SLNs from patients clinically diagnosed as lymph node metastasis-negative. Therefore, the HEVs may function as blood-carrying vessels to the established metastases in their lymph nodes. However, the role of HEVs in lymphatic spread of OSCC is unclear, and need to be elucidated in future studies.

VEGF-D has been associated with lymph node metastasis in animal models; however, the relationship between VEGF-D and lymphatic metastasis is controversial: for example, VEGF-D is down-regulated in some types of carcinoma tissue, such as colorectal cancer and lung adenocarcinoma [34,35]. In the current study, VEGF-D expression was significantly correlated with progression of the nodal status. However, neither VEGF-A nor VEGF-C expression was significantly correlated with the progression of lymph node metastasis. Our results suggest that VEGF-D expression is a promising predictive factor for lymph node metastasis, and that tumor-derived VEGF-D plays physiological roles distinct from VEGF-A and VEGF-C in lymph node metastasis in OSCC. Tanaka et al. reported that the VEGF-D/VEGFR3 autocrine mechanism regulates tumor cell proliferation and inhibition of apoptosis in gastric carcinoma [36]. We also found that lymph node metastasis was significantly progressed when both VEGF-D and VEGFR3 were High in tumor cells at the primary site. Further studies are needed to identify the VEGF-D/VEGFR3-associated autocrine loop responsible for the lymphatic spread of cancer cells in OSCC.

In summary, we showed that SLN lymphangiogenesis occurs even before metastasis. We showed that VEGF-A and VEGF-D play a critical role in this process in OSCC. VEGF-D is also a potential predictive marker of positive lymph node metastasis in cN0 patients. Although we showed that HEVD is increased by VEGF-A from the primary tumor, the role of HEVs was not lymphvascular niche formation. The role of HEVs in the metastatic process of OSCC should also be clarified in future studies. Finally, the inclusion of a therapeutic approach to block lymphangiogenic factors, such as VEGF-D, may be beneficial to prevent the lymphatic spread of tongue cancer with intense intranodal lymphangiogenesis.
